# Association of Age at Cancer Diagnosis and Clinical Trial Participation

**DOI:** 10.1001/jamanetworkopen.2020.37573

**Published:** 2021-02-18

**Authors:** Jessica Keim-Malpass, Héctor E. Alcalá

**Affiliations:** 1 Department of Acute and Specialty Care, University of Virginia School of Nursing, Charlottesville; 2 Department of Pediatrics, University of Virginia School of Medicine, Charlottesville; 3 Program in Public Health, Department of Family, Population, and Preventive Medicine, Renaissance School of Medicine, Stony Brook University, Stony Brook, New York

## Abstract

This cross-sectional study uses data from the 2016 Behavioral Risk Factor Surveillance System data set to examine the association of age at cancer diagnosis with patients’ participation in clinical trials among cancer survivors.

## Introduction

Overall survival outcomes for adolescents and young adults with cancer have not kept pace with those of their younger childhood or older adult counterparts. Although the causes for this disparity are multifactorial, 1 prominent reason may be the unequal access to clinical trials.^1,2^ Numerous studies have shown a survival advantage for children who enrolled in clinical trials for various types of malignant neoplasms,^3^ yet there is unequal representation in clinical research among older adolescents and young adults.^2^ It is estimated that only 10% of adolescents aged 15 to 19 years and approximately 1% of young adults aged 20 to 30 years enter clinical trials of pediatric or adult cooperative groups.^2^ On the opposite end of the age spectrum, older adults represent more than half of all patients with cancer diagnoses, and previous reports suggest that they, too, are underrepresented in cancer clinical trials. Previous studies demonstrate that patients older than 65 years account for less than 10% of all patients enrolled in clinical trials sponsored by the National Cancer Institute.^4^ Our analysis sought to examine the association of age at diagnosis with cancer-related clinical trial participation among a nationally representative sample of cancer survivors.

## Methods

The data source for this cross-sectional study was the 2016 Behavioral Risk Factor Surveillance System data set.^5^ The Behavioral Risk Factor Surveillance System is a nationally representative survey of noninstitutionalized adults whose responses are collected by both landline and cell phone interviews. In 2016, a cancer survivorship module was included in the core outcomes that elicited additional responses among respondents who indicated they had a past or current cancer diagnosis. Age at cancer diagnosis was the primary risk factor. Other independent variables were controlled for in the multivariable model, including race/ethnicity, sex, educational attainment, income, current insurance status, and cancer type. The dichotomous (yes/no) dependent variable was enrollment in a clinical trial that was elicited from the question “Did you participate in a clinical trial as a part of your cancer treatment?” This report followed the Strengthening the Reporting of Observational Studies in Epidemiology (STROBE) reporting guideline. Approval for this study was waived by the University of Virginia institutional review board because deidentified publicly available data were used in this analysis.

Univariate statistics were run for all study variables, and all statistics except sample sizes were weighted. Logistic regression models were then fit to estimate the odds of participating in a clinical trial as part of cancer treatment. A final multivariable model included all confounders simultaneously, along with age. Analyses were conducted from November 15 and December 21, 2020, using Stata version 14.1 software (StataCorp LLC).

## Results

Only 345 patients (5.98%) of the total number in the study sample (N = 5922) indicated that they ever participated in a clinical trial related to their cancer care. In the full cohort, 2175 (38.79%) were males, 3747 (61.21%) were females, 5510 (91.50%) were White, and 5876 (98.61%) were not Hispanic or Latino. Additional patient characteristics are given in Table 1. Most respondents (3169 [53.31%]) were between the ages of 40 and 64 years at the time of their cancer diagnosis.

**Table 1.** Sample Characteristics for 5922 Cancer Survivors From the Behavioral Risk Factor Surveillance System^a^

Characteristic	Full sample, No. (%) [SE]	Participation in clinical trials, No. (%) [SE]	
		Yes (n = 345)	No (n = 5577)
Participation in a clinical trial for cancer treatment			
No	5577 (94.02) [0.47]		
Yes	345 (5.98) [0.47]		
Race			
White	5510 (91.50) [0.69]	304 (86.61) [3.24]	5206 (91.82) [0.70]
Black	228 (5.82) [0.63]	30 (11.68) [3.22]	198 (6.45) [0.63]
Other^b^	184 (2.67) [0.32]	11 (1.7) [0.64]	173 (2.74) [0.34]
Hispanic or Latino ethnicity			
No	5876 (98.61) [0.29]	341 (98.52) [1.25]	5535 (98.62) [0.30]
Yes	46 (1.39) [0.29]	4 (1.48) [1.25]	42 (1.36) [0.30]
Sex			
Male	2175 (38.79) [0.98]	131 (45.84) [4.09]	2044 (38.34) [1.01]
Female	3747 (61.21) [0.98]	214 (54.16) [4.09]	3533 (61.66) [1.01]
Educational level			
>High school completion	3902 (60.35) [1.02]	228 (57.36) [4.14]	3674 (60.54) [1.05]
≤High school completion	2020 (39.65) [1.02]	117 (42.64) [4.14]	1903 (39.46) [1.05]
Income, $			
<50 000	2892 (48.16) [1]	169 (50.86) [4.03]	2723 (47.99) [1.03]
≥50 000	2269 (39.53) [0.96]	140 (39.51) [3.88]	2129 (39.53) [0.99]
Missing	761 (12.31) [0.65]	36 (9.63) [2.18]	725 (12.48) [0.68]
Current age, y			
<65	2093 (47.66) [1.01]	141 (55.8) [3.88]	1952 (47.15) [1.04]
≥65	3829 (52.34) [1.01]	204 (44.2) [3.88]	3625 (52.85) [1.04]
Age at diagnosis, y			
<18	79 (2.57) [0.40]	10 (7.87) [3.34]	69 (2.23) [0.36]
18-39	849 (19.36) [0.92]	45 (17.97) [3.23]	804 (19.45) [0.95]
40-64	3169 (53.17) [1.00]	218 (63.81) [4.04]	2951 (52.49) [1.03]
≥65	1825 (24.91) [0.80]	72 (10.34) [1.62]	1753 (25.83) [0.84]
Insured			
No	146 (4.02) [0.52]	9 (3.99) [1.62]	137 (4.02) [0.54]
Yes	5776 (95.98) [0.52]	336 (96.01) [1.62]	5440 (95.98) [0.54]
Cancer type^c^			
Solid tumors	5385 (90.95) [0.56]	294 (80.71) [3.85]	5091 (91.6) [0.53]
Hematologic malignant neoplasms	187 (3.14) [0.33]	25 (9.68) [2.63]	162 (2.72) [0.31]
Central nervous system, brain, and other cancers	350 (5.91) [0.46]	26 (9.61) [3.26]	342 (5.67) [0.44]

^a^ All statistics except sample size are weighted.

^b^ Other races included American Indian or Alaska Native, Asian, Native Hawaiian or Pacific Islander, and other race or multiracial.

^c^ Nonmelanoma skin cancer survivors were excluded from analyses.

Table 2 shows the bivariate associations between the variables and clinical trial participation along with the results from the final multivariable model. Adolescents and young adults between 18 and 39 years and adults 65 years or older at the time of their cancer diagnosis had significantly lower odds of clinical trial participation (adjusted odds ratio, 0.38; 95% CI, 0.15-0.89 for 18- to 39-year-olds; adjusted odds ratio, 0.14; 95% CI, 0.06-0.35 for adults ≥65 years). Patients with hematologic malignant neoplasms were significantly more likely to participate compared with those with other cancer types (adjusted odds ratio, 3.72; 95% CI, 1.99-6.97).

**Table 2.** Odds of Participating in Clinical Trial for Treatment Among 5922 Cancer Survivors From the Behavioral Risk Factor Surveillance System

Characteristic	OR (95% CI)	AOR (95% CI)
Race		
White	1 [Reference]	1 [Reference]
Black	2.27 (1.18-4.39)^a^	2.06 (1.16-3.66)^a^
Other^b^	0.66 (0.30-1.45)	0.52 (0.20-1.38)
Hispanic or Latino ethnicity		
No	1 [Reference]	1 [Reference]
Yes	1.07 (0.19-6.09)	1.09 (0.17-6.80)
Sex		
Male	1 [Reference]	1 [Reference]
Female	0.73 (0.53-1.03)	0.73 (0.52-1.01)
Educational attainment		
>High school completion	1 [Reference]	1 [Reference]
≤High school completion	1.14 (0.81-1.61)	1.19 (0.83-1.71)
Income, $		
<50 000	1 [Reference]	1 [Reference]
≥50 000	0.94 (0.67-1.33)	0.95 (0.65-1.38)
Missing	0.73 (0.43-1.24)	0.86 (0.51-1.48)
Current age, y		
<65	1 [Reference]	1 [Reference]
≥65	0.71 (0.51-0.97)^a^	1.11 (0.77-1.60)
Age at diagnosis, y		
<18	1 [Reference]	1 [Reference]
18-39	0.26 (0.09-0.73)^a^	0.38 (0.15-0.89)^a^
40-64	0.34 (0.13-0.90)^a^	0.49 (0.22- 1.09)
≥65	0.11 (0.04-0.31)^a^	0.14 (0.06- 0.35)^a^
Insured		
No	1 [Reference]	1 [Reference]
Yes	1.01 (0.42-2.42)	1.04 (0.43-2.52)
Cancer type^c^		
Solid tumors	1 [Reference]	1 [Reference]
Hematologic malignant neoplasms	4.04 (2.15-7.59)^a^	3.72 (1.99-6.97)^a^
Central nervous system, brain, and other cancers	1.92 (0.90-4.08)	1.69 (0.95-3.02)

Abbreviations: AOR, adjusted odds ratio; OR, odds ratio.

^a^ Indicates a significant association.

^b^ Other races included American Indian or Alaska Native, Asian, Native Hawaiian or Pacific Islander, and other race or multiracial.

^c^ Nonmelanoma skin cancer survivors were excluded from analyses.

## Discussion

This nationally representative analysis highlights the 2 age groups at great risk for unequal participation in cancer clinical trial: (1) adolescents and young adults and (2) older adults. Several limitations of this analysis must be addressed. First, it was not possible to ascertain if the study participants were eligible or invited to participate in clinical trials during their cancer treatment. Second, the sampling strategies used in the Behavioral Risk Factor Surveillance System may limit the generalizability of the study findings. Third, the cancer survivors included are predominantly long-term survivors and may not represent patients with more advanced cancer stages at diagnosis. The current clinical trial infrastructure must expand and modernize eligibility criteria for trials and continue to promote innovation in enrollment and retention among patients that mirror real-world clinical populations.^6^
